# Experimental and Emerging Free Fatty Acid Receptor Agonists for the Treatment of Type 2 Diabetes

**DOI:** 10.3390/medicina58010109

**Published:** 2022-01-11

**Authors:** Angelo Maria Patti, Rosaria Vincenza Giglio, Nikolaos Papanas, Dragos Serban, Anca Pantea Stoian, Kalliopi Pafili, Khalid Al Rasadi, Kanya Rajagopalan, Ali A. Rizvi, Marcello Ciaccio, Manfredi Rizzo

**Affiliations:** 1 Department of Health Promotion, Mother and Child Care, Internal Medicine and Medical Specialties, School of Medicine, University of Palermo, 90133 Palermo, Italy; pattiangelomaria@gmail.com (A.M.P.); manfredi.rizzo@unipa.it (M.R.); 2 Department of Biomedicine, Neuroscience, and Advanced Diagnostics, Institute of Clinical Biochemistry, Clinical Molecular Medicine and Laboratory Medicine, University of Palermo, 90127 Palermo, Italy; giglio.rosaria.vincenza@gmail.com (R.V.G.); marcello.ciaccio@unipa.it (M.C.); 3 Diabetes Centre, Second Department of Internal Medicine, Democritus University of Thrace, 68132 Alexandroupolis, Greece; papanasnikos@yahoo.gr (N.P.); kpafili@hotmail.com (K.P.); 4 Forth Surgery Department, Faculty of Medicine, Carol Davila University, 050098 Bucharest, Romania; dragos.serban@umfcd.ro; 5 Department of Diabetes, Faculty of Medicine, Nutrition and Metabolic Diseases, Carol Davila University, 050474 Bucharest, Romania; ancastoian@yahoo.com; 6 Medical Research Center, Sultan Qaboos University, Muscat 123, Oman; khalid77@squ.edu.om; 7 Department of Medicine, University of Central Florida College of Medicine, Orlando, FL 32827, USA; Kanya.Rajagopalan@med.ucf.edu; 8 Division of Endocrinology, Diabetes and Metabolism, University of South Carolina School of Medicine, Columbia, SC 29208, USA; 9 Department of Laboratory Medicine, University Hospital, 90127 Palermo, Italy

**Keywords:** cardiovascular risk, free fatty acids, Type 2 diabetes, metabolism, GLP-1, incretins

## Abstract

The current management of Type 2 Diabetes Mellitus (T2DM) includes incretin-based treatments able to enhance insulin secretion and peripheral insulin sensitivity as well as improve body mass, inflammation, plasma lipids, blood pressure, and cardiovascular outcomes. Dietary Free Fatty Acids (FFA) regulate metabolic and anti-inflammatory processes through their action on incretins. Selective synthetic ligands for FFA1-4 receptors have been developed as potential treatments for T2DM. To comprehensively review the available evidence for the potential role of FFA receptor agonists in the treatment of T2DM, we performed an electronic database search assessing the association between FFAs, T2DM, inflammation, and incretins. Evidence indicates that FFA1-4 agonism increases insulin sensitivity, induces body mass loss, reduces inflammation, and has beneficial metabolic effects. There is a strong inter-relationship between FFAs and incretins. FFA receptor agonism represents a potential target for the treatment of T2DM and may provide an avenue for the management of cardiometabolic risk in susceptible individuals. Further research promises to shed more light on this emerging topic.

## 1. Introduction

Free Fatty Acids (FFA) are energy substrates that play an important role in various biological processes. FFAs are distinguished based on their chain length. Short-Chain Fatty Acids (SCFA) have 1–6 carbon atoms, Medium-Chain Fatty Acids (MCFA) possess 7–12 carbon atoms, while those with more than 12 carbon atoms are designated as Long-Chain Fatty Acids (LCFAs) [1,2]. Medium- and Long-Chain FFAs are derived from dietary triglycerides, while Short-Chain Fatty Acids (SCFA) are produced by intestinal microbial fermentation of indigestible dietary fiber [1]. FFAs also serve as natural ligands for a group of orphan G Protein-Coupled Receptors (GPCRs) called Free Fatty Acid Receptors (FFARs), which intertwine metabolism and immunity via the regulation of inflammation and the secretion of peptide hormones [3].

Several FFARs activated by FFAs of various chain lengths have been identified and characterized. FFAR1 (GPR40) and FFAR4 (GPR120) are activated by long-chain saturated and unsaturated fatty acids, while FFAR3 (GPR41) and FFAR2 (GPR43) are activated by acetate, butyrate, and propionate [3]. Synthetic ligands, selective for FFA1-4 receptors, have been developed as potential treatments for Type 2 Diabetes Mellitus (T2DM). Fasiglifam, an FFA1 agonist, has been shown to improve glycemic control and reduce Hemoglobin A1c (HbA1c) levels in patients with T2DM, without an increased risk of hypoglycemia [4]. FFA4 agonism increases insulin sensitivity, induces body mass loss, and reduces inflammation. The metabolic and anti-inflammatory effects of SCFA are linked to the activation of FFA2 and FFA3 [3,4].

T2DM and its related complications represent a major global health challenge with an increasing impact due to the current COronaVIrus Disease-19 (COVID-19) pandemic [5,6]. There is a need for novel approaches in the treatment of T2DM, particularly with COVID-19 as a persistent accentuating factor [7,8,9]. We performed an electronic database search (MEDLINE, EMBASE, and SCOPUS) assessing the association between FFA, T2DM, inflammation, and incretin-based therapies. Our aim was to provide a comprehensive review of available evidence on the potential role of FFAR agonists (FFA-RAs) in the treatment of T2DM.

## 2. Free Fatty Acids, Glucose Metabolism, and Type 2 Diabetes

Dietary FFAs, including omega-3 fatty acids have been shown to modulate the metabolic and inflammatory processes associated with T2DM [2]. Many of the biological effects of FFAs have been attributed to GPCRs; the most characterized FFARs are the two LCFA-specific ones, FFA1 and FFA4, and the SCFA-specific receptors FFA2 and FFA3. FFAR agonism has been shown to have beneficial metabolic actions, and drug development selective to FFA agonists has potential benefits as novel clinical treatments for T2DM [2]. The omega-3 fatty acids obtained from fish oils and the SCFAs derived from the fermentation of dietary fiber have effects on the metabolic and inflammatory processes associated with obesity and T2DM, which are attributable to the activation of FFA1-4 receptors [10].

FFA1 receptor agonism enhances glucose-stimulated insulin secretion from the pancreas and complete agonists of this receptor increase incretin release from the intestine, thereby enhancing pancreatic insulin secretion and promoting satiety [11]. FFA4 agonism has an anti-inflammatory effect on macrophages, which can improve systemic sensitivity to insulin, while also additionally releasing incretin from the intestine [1]. FFA2 and FFA3 receptors are linked to the beneficial metabolic effects associated with probiotics in the intestine, with the release of incretin from entero-endocrine cells and both systemic anti-inflammatory and pro-inflammatory effects [12].

### 2.1. Free Fatty Acids 1 (FFA1)

FFA1 are activated by various saturated, monounsaturated, and long-chain polyunsaturated FFAs and are coupled with Gq/11 expressed in pancreatic beta cells (associated with increased Glucose-Stimulated Insulin Secretion—GSIS) [13], and alpha cells producing glucagon within the pancreas [14]. FFA1 is expressed by enteroendocrine cells by regulating the release of incretin hormones, such as Glucagon-Like Peptide-1 (GLP-1) and Cholecystokinin (CCK) [15]. The observed effects of FFA1 on pancreatic beta cell viability have been controversial, with pancreatic-specific FFA1 overexpression associated with disrupted islet morphology and impaired beta cell function, while FFA1 disruption being linked to increased viability of beta cells in mice fed a high-fiber diet (HFD) [16].

**Figure 1:**
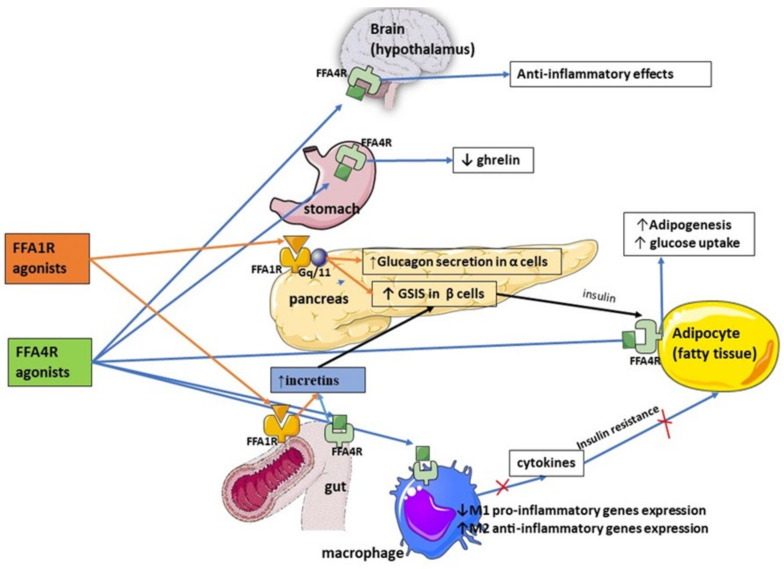
Potential actions of free fatty acids 1 receptor (FFA1R) and free fatty acids 4 receptor (FFA4R) agonists.

The ability of synthetic FFA1 agonists to induce incretin release depends on whether the compound is a partial or complete agonist [17]. Patients treated with fasiglifam, an orally available FFA1 agonist [18], have shown reduced blood glucose levels, increased insulin levels, and lower HbA1c levels [19], with significantly fewer side effects than traditional glucose-lowering therapies. There were no significant increases in body mass, with a concomitant reduction in hypoglycemia [19], insulin resistance, and glucagon secretion in response to fasiglifam treatment in patients with T2DM [20,21]. A diagrammatic summary of the actions of FFAR agonists is depicted in Figure 1.

### 2.2. Free Fatty Acids 4 (FFA4)

FFA4 is expressed in enteroendocrine cells, lungs, brain, white adipose tissue, heart, and liver [2]. Within adipose tissue, the activation of FFA4 in adipocytes is associated with an increase in adipogenesis and glucose uptake [22], promoting adiposity and obesity. FFA4 agonism is associated with improved insulin sensitivity [23]. Increased insulin secretion, satiety, and improved glycemic control have been attributed to FFA4-dependent release of GLP-1 from entero-endocrine cells. FFA4 also co-localizes with ghrelin, inhibiting its secretion [24]. Reduced levels of FFA4 mRNA have been found in the pancreatic islets of individuals with diabetes and prediabetes, demonstrating an attenuation of the protective effects of ω-3 fatty acids, such as eicosapentaenoic acid against palmitate-induced cell apoptosis [25], and inhibition of glucose-dependent somatostatin release and regulation of glucagon secretion [26].

FFA4 expression in macrophages increases in response to obesity. The anti-inflammatory effects are largely associated with FFA4-mediated recruitment of β-arrestin 2, activation of FFA4 reduced pro-inflammatory gene expression in M1 macrophages, and increased expression of M2 anti-inflammatory genes with reduced tissue macrophage infiltration of adipocytes [27]. FFA4 has been associated with the anti-inflammatory effects of ω-3 and ω-9 fatty acids in the hypothalamus, reducing diet-induced inflammation and body adiposity [28]. Thus, they have great potential for the treatment of metabolic diseases. However, despite their positive pleiotropic effects, there is a paucity of studies in the clinical use of FFA4 agonists. [29].

## 3. The Relationship between Free Fatty Acids and Incretins

Incretin-based therapies with GLP-1 Receptor Agonists (RAs) and Dipeptidyl Peptidase-4 (DPP-4) inhibitors improve insulin secretion and/or peripheral insulin sensitivity in patients with T2DM [30,31,32]. The inter-relationship between FFAs and incretins, as well as the physiologic roles of FFA receptors, remain to be fully elucidated. The clinical pharmacology of FFAs and challenges in their use as potential therapeutic options for T2DM are currently under extensive investigation [33,34].

The beneficial effects mediated by SCFA on body mass and glucose homeostasis are due to the increased secretion of incretins, such as GLP-1, Glucose-dependent Insulinotropic Polypeptide (GIP), and Peptide YY (PYY), through mechanisms partly dependent on FFA2/FFA3 [35]. It is hoped that the development of more potent and selective FFA2 and FFA3 agonists will facilitate elucidation of the metabolic effects of FFA2 and FFA3 and provide future treatments for T2DM. The role of the FFARs in improving glucose uptake, decreasing colon motility and contractility, increased GLP-1 secretion, and inhibition of leukocyte activation is being investigated [36]. Although a series of selective compounds are on the horizon, their clinical use has been limited due to low solubility and poor pharmacokinetics [37]. Table 1 shows the prominent actions of FFAs on glucose metabolism and the incretin pathway.

## 4. Therapeutic Avenues

In a double-blind, randomized phase 1 study in patients with T2DM, fasiglifam showed a significant lowering in fasting glucose from baseline (up to 93 mg/dL), while the post-challenge glucose during an Oral Glucose Tolerance Test (OGTT) fell by an average of 172 mg/dL) [38]. In a larger phase 2 T2DM study, fasiglifam at a dose of 50–200 mg revealed a blood glucose lowering effect similar to that of glimepiride 4 mg, showing a decrease in HbA1c of approximately 1%. In both studies, hypoglycemic events during treatment were similar in the fasiglifam and placebo groups (2% vs. 3%), but lower than in the comparator group treated with glimepiride (19%) [39]. In another phase 3 study lasting 24 weeks, the mean changes of HbA1c from baseline in the placebo group (0.16%), and in the groups receiving fasiglifam at 25 mg (−0.57%) and fasiglifam at 50 mg (−0.83%) suggested that the latter held great potential for the treatment of T2DM [40]. Unfortunately, there are no other studies in the literature in diabetic patients treated with FFA-RA; however, pivotal studies in animals demonstrate similar effects to the findings mentioned above.

Several hypothetical avenues have also been proposed for the development of clinical treatments based on FFA receptor agonism, including the co-therapeutic approaches involving FFA receptor agonists and current T2DM therapies. AS2575959, an FFA1 agonist, acts synergistically with a DPP-IV inhibitor to improve glucose homeostasis [41]; Fasiglifam and metformin showed enhanced antidiabetic effects in association with each other [42]; DS-1558, an FFA1 agonist, acts synergistically with exendin-4 to improve glucose homeostasis in diabetic mice [43]; metabolic effects with dapagliflozin plus long-acting exenatide (LAR) include less FFA suppression than placebo during an oral glucose tolerance test (OGTT), suggesting compensatory lipid mobilization for energy production when glucose availability was reduced due to glucosuria [44].

In a similar fashion, the GLP-1RA liraglutide reduces fat-induced lipid formation and exhibits a protective effect against lipotoxicity-induced oxidative stress [45]. The latter seems to have a significant relevance, since liraglutide has the potential to reduce oxidative stress in T2DM after only few weeks of treatment [46]. In addition, liraglutide can quickly reduce atherogenic small, dense LDL particles in such patients [47,48], while some traditional anti-antidiabetic agents and even few statins did not show a similar favorable effect on LDL subclass distribution [49,50]; yet, for statins, the duration of therapy is critical in order to show full cardio-renal benefit [51,52]. These observations have led to the hypothesis that liraglutide and other incretin-based drugs have a direct anti-atherogenic action in T2DM, reducing atherosclerosis formation and progression [46], which in turn could be one the main underlying mechanisms explaining the beneficial effect of these novel antidiabetic agents on cardiovascular events and mortality in type-2 diabetic patients [53]. These actions augment those of FFAs, and a synergistic effect is expected, though not proven, when both are used in combination.

Regulation of FFA in relation to metabolic factors were also investigated in drug naive subjects with T2DM who were treated with 50–100 mg of canagliflozin, a sodium–glucose transporter-2 agent, as monotherapy for 3 months [54]. Metabolic parameters were compared between groups with low FFA and high FFA. Groups with higher FFA had greater degrees of HbA1c reduction and increases in insulin, along with significant reductions of non-HDL-C, UA, and adipo-IR. Body mass and whole-body insulin resistance were also decreased in those with elevated FFA, though better glycemic control along with increased beta cell function and decreased atherogenic cholesterol were seen in those with reduced FFA [54].

The relationship between FFAs and diabetes is in the process of being elucidated. The plasma of patients with T2DM show an increased concentration of atherogenic small, dense low-density lipoproteins (sdLDL), placing them at higher cardiovascular risk [55,56,57] particularly in those populations where there is a strong genetic heritage [58,59,60]. These lipoprotein particles are easily oxidized in the arterial intima [61], initiating a key pathophysiologic mechanism in the atherosclerotic cascade that is enhanced in the presence of inflammation and altered endothelium [46]. Notably, in patients with T2DM, endothelial dysfunction, inflammation, and atherosclerosis are closely linked to changes in cytokine biomarkers [62,63] and increased sdLDL [64].

In a recent study, Ha and colleagues reported that FFAs and docking protein 1 (DOK1) were associated with insulin resistance in patients with T2DM, even in the absence of obesity and prediabetes [65]. They speculated that DOK1 downregulation might inhibit lipid synthesis, thus induce lipolysis and possibly worsening insulin resistance. These complex metabolic pathways represent fertile targets for innovative therapeutic interventions, possibly involving the use of FFAR agonists. Key safety monitoring processes in clinical trials were able to detect the rare but serious liver toxicity signals; however, leading to timely termination [66,67]. Table 2 shows a summary of seminal trials evaluating the effectiveness of FFA receptor agonists alone and in combination with other glucose-lowering agents.

## 5. Conclusions

Evidence to-date indicates that the development of treatments based on FFARs agonism could provide a new and novel way to treat T2DM. Further studies may assess whether there are relationships between physical activity (e.g., resistance training, high-intensity interval training, or aerobics) and FFA1-4; since the evidence indicates its effects on insulin sensitivity and inflammation, this may represent another line of research. However, it is imperative that the safety profile of FFAR agonists be fully evaluated. As a case in point, the clinical trial program of fasiglifam has been terminated due to liver toxicity [66,67]. Pre-clinical and clinical studies with these agents are, therefore, essential. Studies conducted in diabetic patients with features of metabolic syndrome, such as obesity, insulin resistance, and dyslipidemia, will also assess the efficacy and safety of these agents in subjects in need of cardiometabolic risk reduction.

## Figures and Tables

**Table 1:** Effects of Free Fatty Acids on Glucose and Incretin Metabolism.

FFA1 expressed by enteroendocrine cells regulates release of incretin hormones, such as GLP-1 and Cholecystokinin-5, which in turn enhance pancreatic insulin secretion and promote satiety
FFA1 agonists (Fasiglifam) have been shown to improve glycemic control, increase insulin sensitivity, induce body mass loss, and reduce inflammation
FFA2 and FFA3 receptors are linked to enhanced incretin secretion from enteroendocrine cells
There is still a paucity of research on inter-relationship between FFAs and incretins, research on FFA2/FFA3 agonists, and the role of FFARs in improving glucose uptake and increasing GLP-1 secretion
FFA4 agonism is associated with improved sensitivity to insulin via anti-inflammatory effect on macrophages through recruitment of β-arrestin 2, increased expression of M2 anti-inflammatory genes, and reduced expression of pro-inflammatory in M1 macrophages
FFA4 inhibits secretion of ghrelin, thus stimulating satiety, promoting incretin release leading to a glucoprotective effect in diabetes, and regulating glucagon secretion
FFAs couple with FFARs to regulate inflammation and peptide hormone secretion

FFA: Free Fatty Acids, FFARs: Free Fatty Acid Receptors, GLP-1: Glucagon-Like Peptide-1.

**Table 2:** Main findings from clinical trials with the use of FFAR agonists.

Trial	Ref.	Year Published	Agent Studied	Study Design	Results
Leifke et al.	[38]	2012	TAK-875	Phase 1, randomized, double-blind, multiple ascending-dose	Significantly lower fasting and post-challenge glucose
Burant et al.	[39]	2012	TAK-875	Phase 2, randomized, double-blind with placebo and glimepiride comparator groups	HbA1c decreased by 1% with study agent
Ito et al.	[42]	2013	TAK-875	Combination with metformin in Zucker diabetic rats	Prevention of diabetes progression and beta-cell dysfunction
Tanaka et al.	[41]	2014	AS2575959	Combination with sitagliptin	Improved glucose homeostasis
Nakashima et al.	[43]	2014	DS-1558	Combination with GLP-1 receptor agonists	Improved glucose tolerance and insulin secretion
Kaku et al.	[40]	2015	TAK-875	Phase 3, randomized, double-blind, placebo-controlled, multiple-dose	Dose-related reduction in HbA1c compared with placebo

FFAR: Free Fatty Acid Receptor, GLP-1: Glucagon-Like Peptide-1, HbA1c: Hemoglobin A1c.
